# Supplementary material for: Temporal Profiles and Dose-Responsiveness of Side Effects with Escitalopram and Duloxetine in Treatment-Naïve Depressed Adults
Source: Behav Sci (Basel). 2018 Jul 17;8(7):64. doi: 10.3390/bs8070064 (PMC6071033; doi:10.3390/bs8070064)
Supplement: Supplementary file 1 [file behavsci-08-00064-s001.pdf]

behavioral sciences

MDPI

Supplementary Data

Temporal Profiles and Dose-Responsiveness of Side Effects with Escitalopram and Duloxetine in Treatment-Naïve Depressed Adults

Philip E. Polychroniou, Helen S. Mayberg, W. Edward Craighead, Jeffrey J. Rakofsky, Ebrahim Haroon, Vivianne Aponte Rivera, Boadie W. Dunlop

Table S1. Selected side effects occurring in <5% of patients

	All patients (n=211)		Escitalopram (n=105)		Duloxetine (n=106)			Escitalopram		Duloxetine		
**Side Effect**	n	%	n	%	n	%	p-value	Mean (days)	SD	Mean (days)	SD	p-value
Appetite increased	10	4.7	8	7.6	2	1.9	0.050	34.8	25.0	43.5	20.5	0.663
Palpitations	8	3.8	2	1.9	6	5.7	0.153	7.0	8.5	39.5	86.5	0.633
Concentration impaired	7	3.3	4	3.8	3	2.8	0.691	30.5	19.7	14.3	13.5	0.281
Irritability	6	2.8	2	1.9	4	3.8	0.414	40.0	53.7	20.0	22.6	0.525
Abnormal dreams	6	2.8	3	2.9	3	2.8	0.991	13.7	8.5	26.3	33.5	0.560
Tremor	5	2.4	3	2.9	2	1.9	0.643	13.3	18.9	10.5	13.4	0.869
Suicidal ideation	3	1.4	2	1.9	1	0.9	0.555	11.0	9.9	1.0	---	0.561
Depression worsening	3	1.4	1	1.0	2	1.9	0.566	8.0	---	15.0	18.4	0.808
Urinary retention	2	0.9	1	1.0	1	0.9	0.995	5.0	---	43.0	---	---
Weight gain	2	0.9	0	0.0	2	1.9	0.157	---	---	141.5	180.3	---
REM sleep behavior	1	0.5	0	0.0	1	0.9	0.318	---	---	3.0	---	---
